# Supplementary figures and images for: Enabling targeted mass drug administration for schistosomiasis in north-western Tanzania: Exploring the use of geostatistical modeling to inform planning at sub-district level
Source: PLoS Negl Trop Dis. 2024 Jan 16;18(1):e0011896. doi: 10.1371/journal.pntd.0011896 (PMC10817176; doi:10.1371/journal.pntd.0011896)

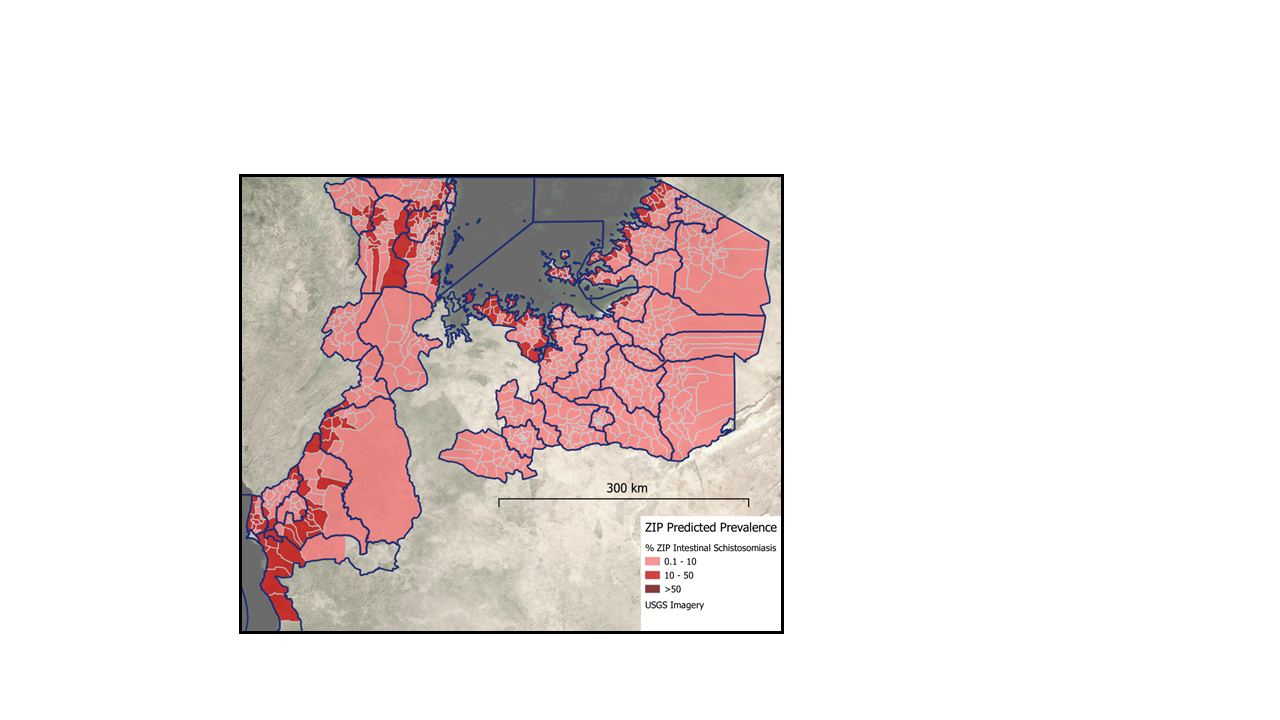

Supplement: S1 Fig — (TIF) [file pntd.0011896.s004.tif]

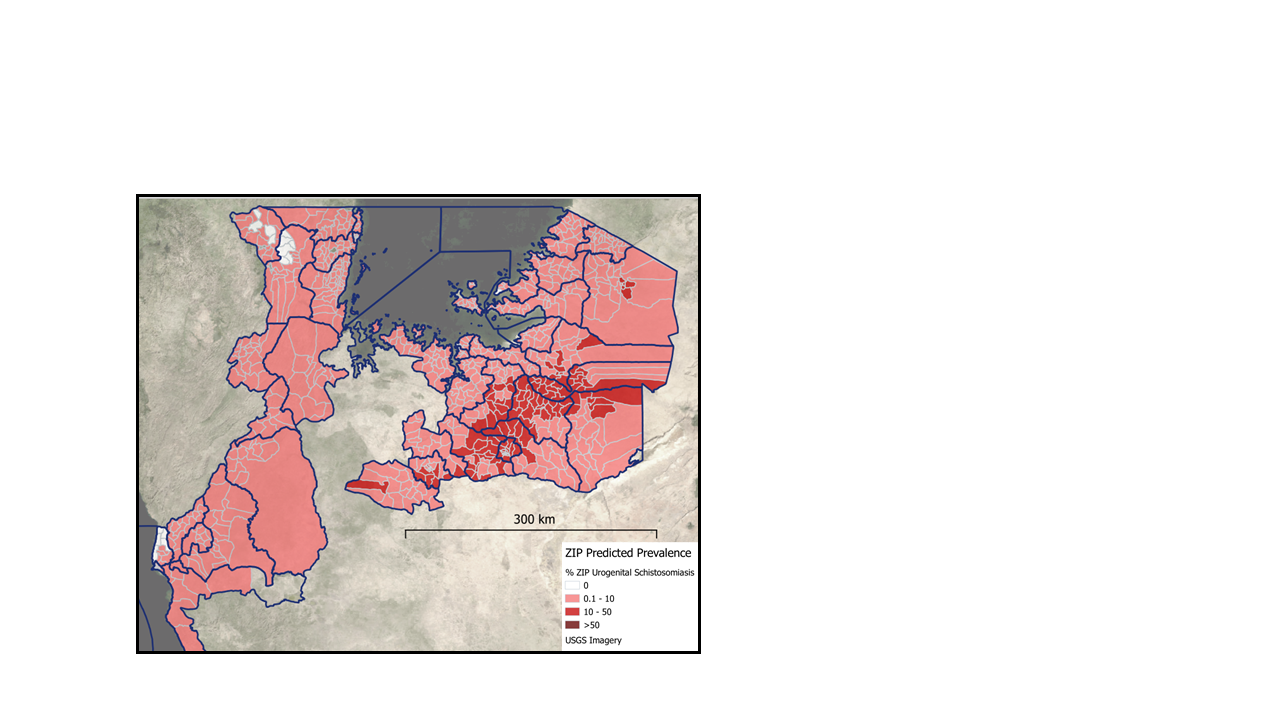

Supplement: S2 Fig — (TIF) [file pntd.0011896.s005.tif]

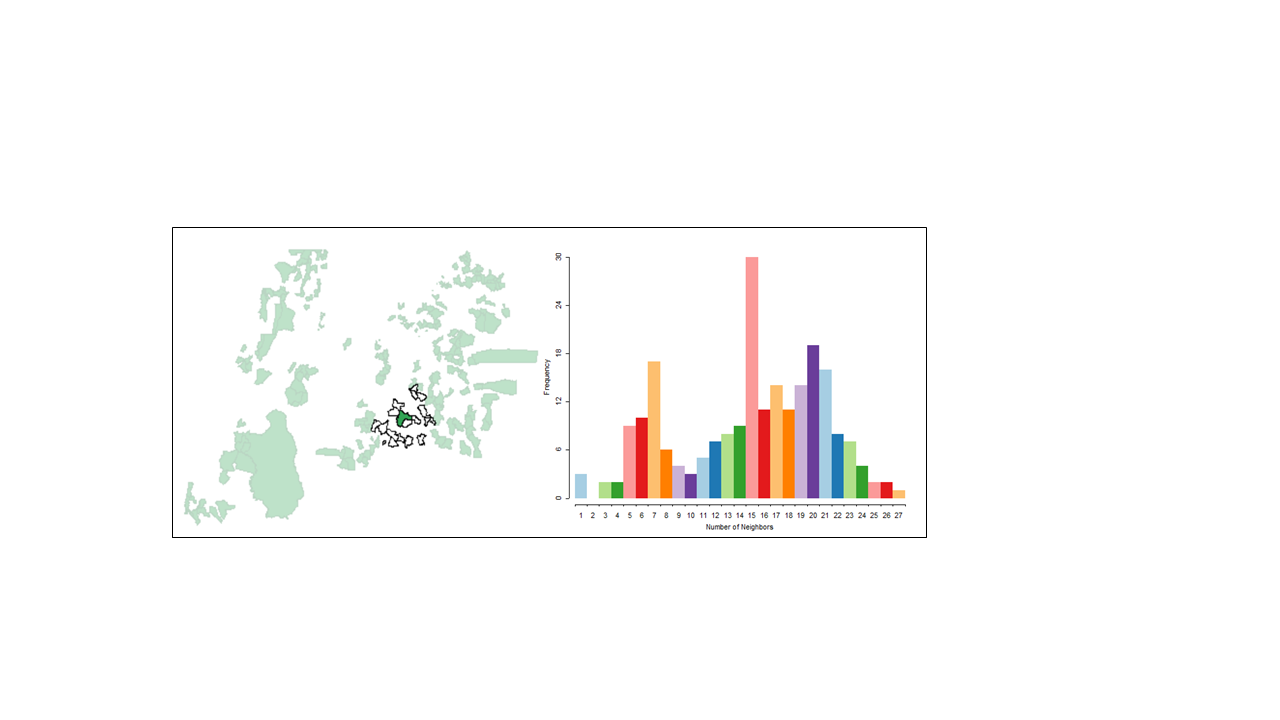

Supplement: S3 Fig — A spatial weights file was generated iteratively to determine the contiguity structure for each ward (threshold: 53597.2m; inverse distance with power: 2; Euclidean distance; queen; order of contiguity: 1st; neighbors: max. 27; min. 1; median: 15). (TIF) [file pntd.0011896.s006.tif]

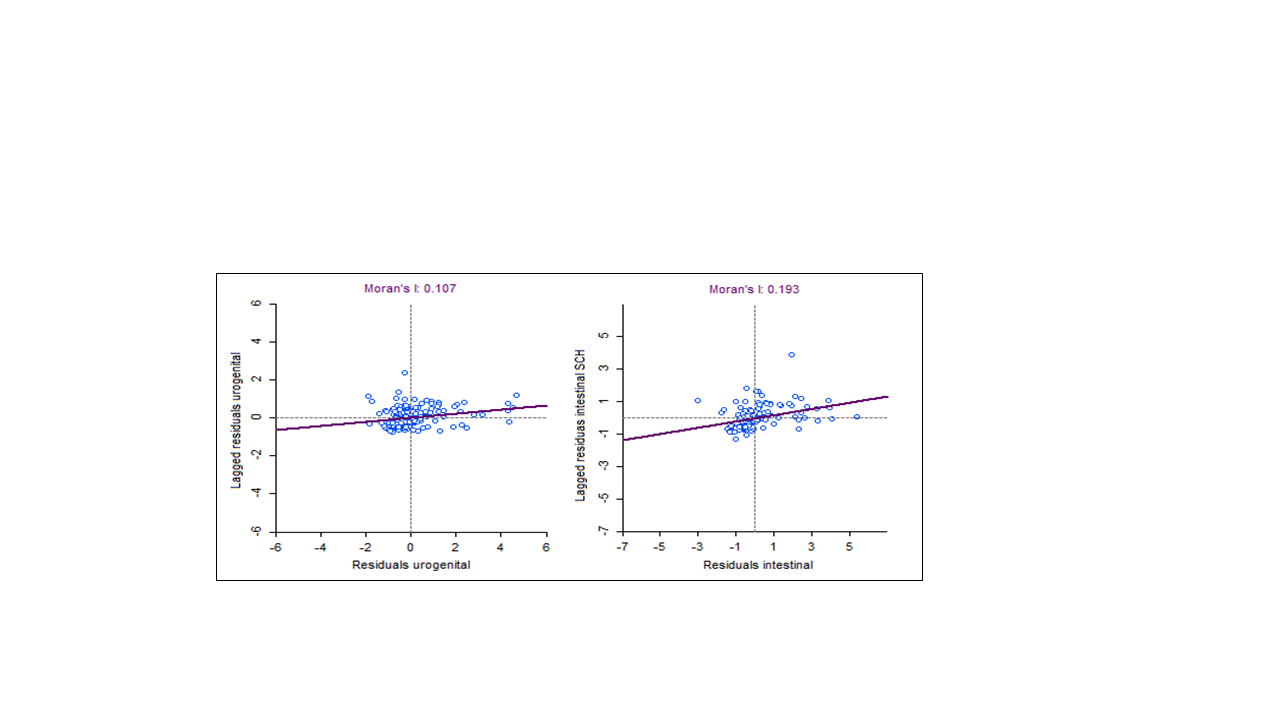

Supplement: S4 Fig — Moran’s I for the ZIP prediction residuals for intestinal (pseudo p-value of p = 0.001) and urogenital (pseudo p-value of p = 0.003) SCH. (TIF) [file pntd.0011896.s007.tif]

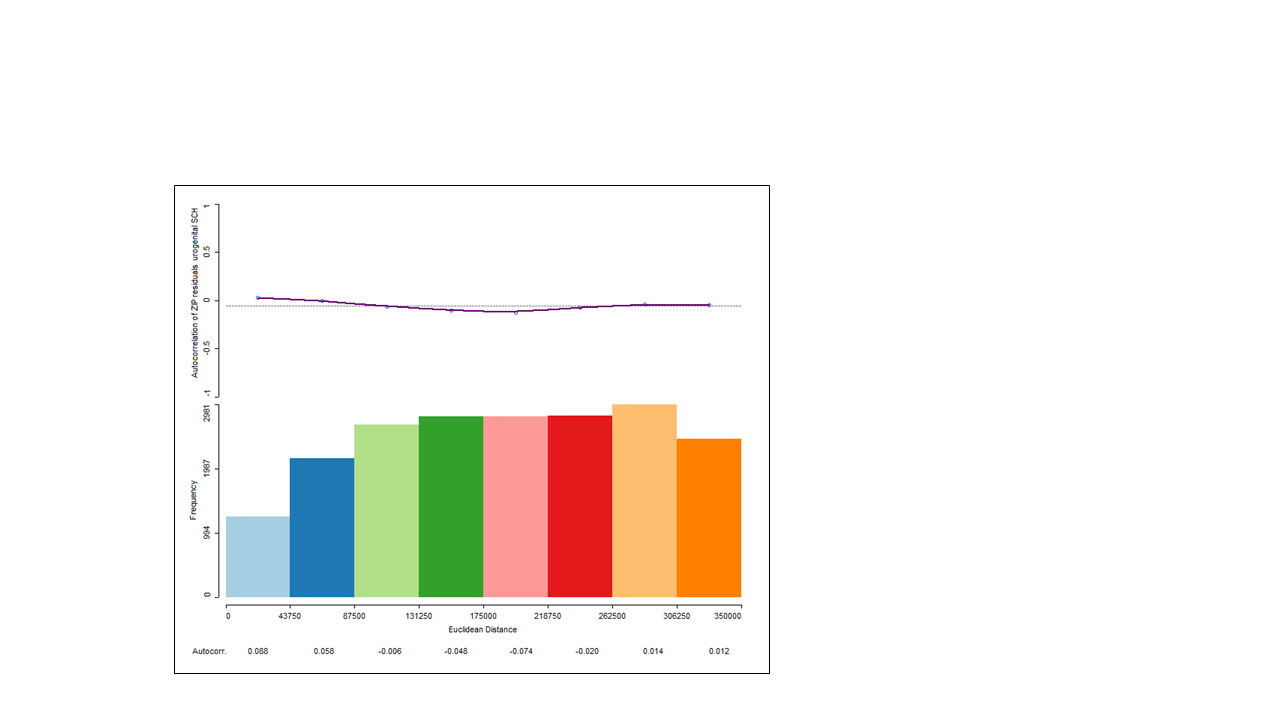

Supplement: S5 Fig — Max. distance = 350km. Zero autocorrelation at 105km. Frequency indicates the number of pairs (total: 19857). (TIF) [file pntd.0011896.s008.tif]

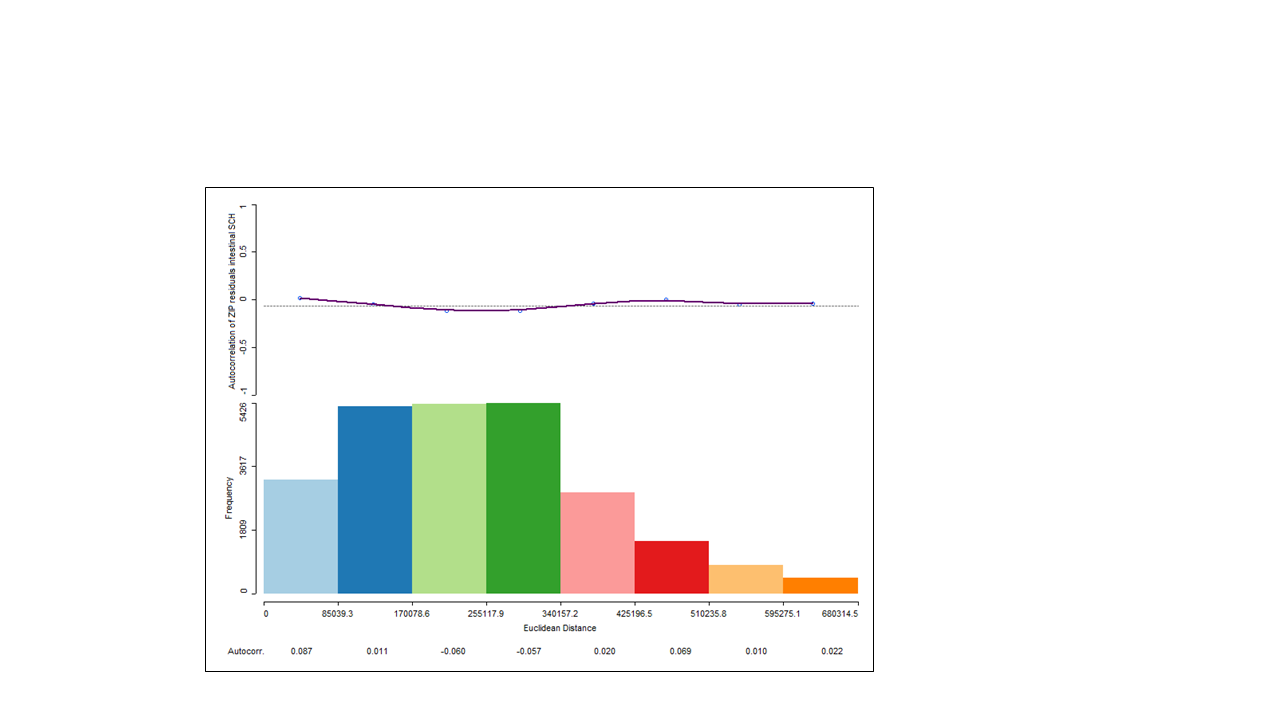

Supplement: S6 Fig — Max. distance = 680km. Zero autocorrelation at 141km. Frequency indicates the number of pairs (total: 24976). (TIF) [file pntd.0011896.s009.tif]

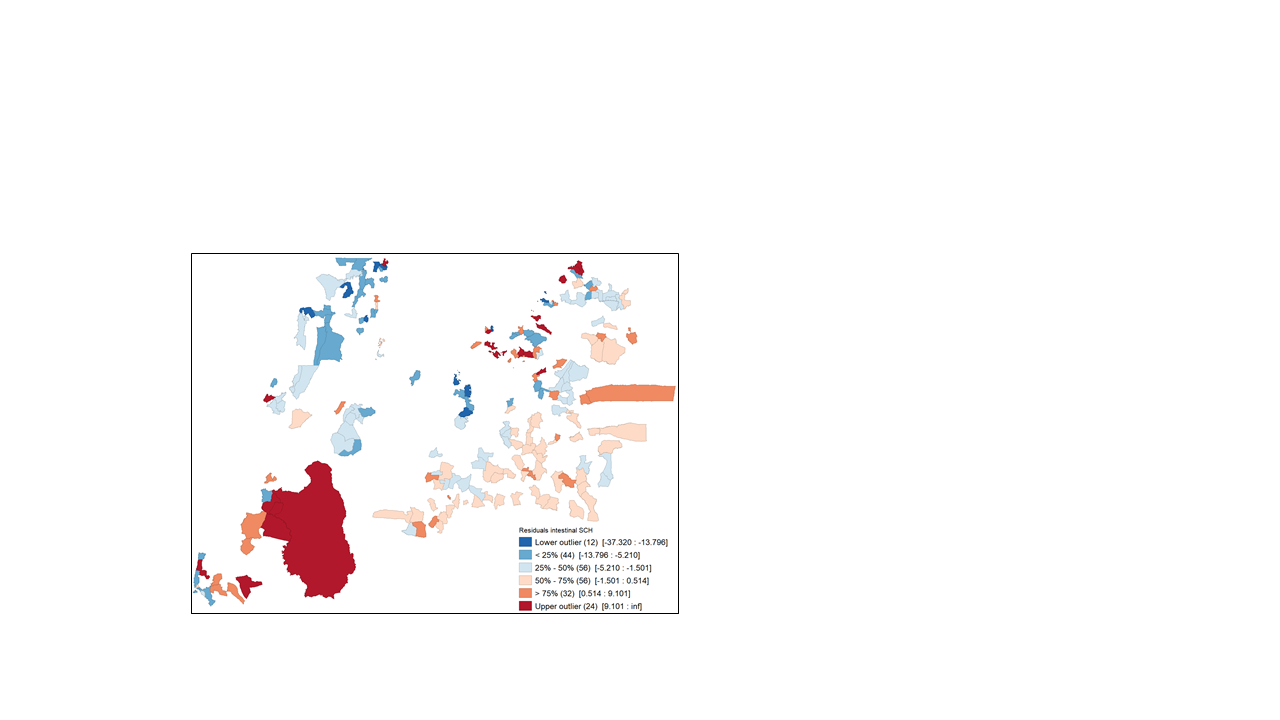

Supplement: S7 Fig — Hinge = 1.5. (TIF) [file pntd.0011896.s010.tif]

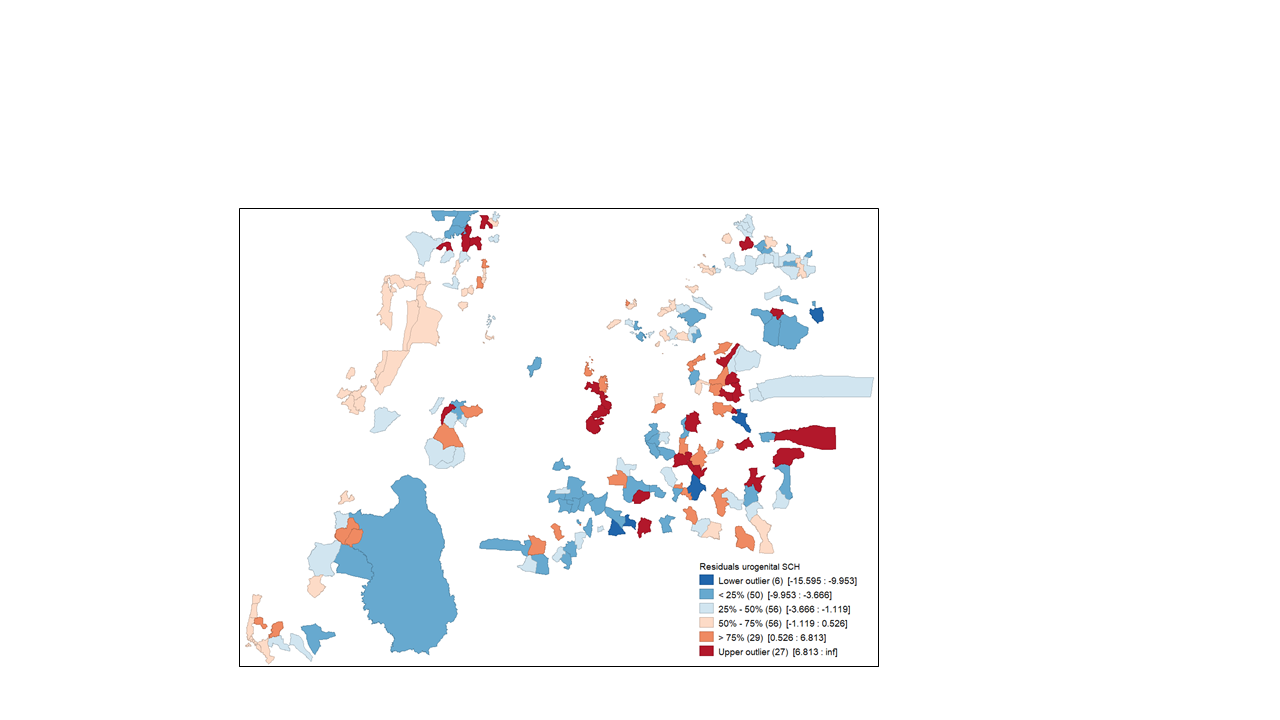

Supplement: S8 Fig — Hinge = 1.5. (TIF) [file pntd.0011896.s011.tif]

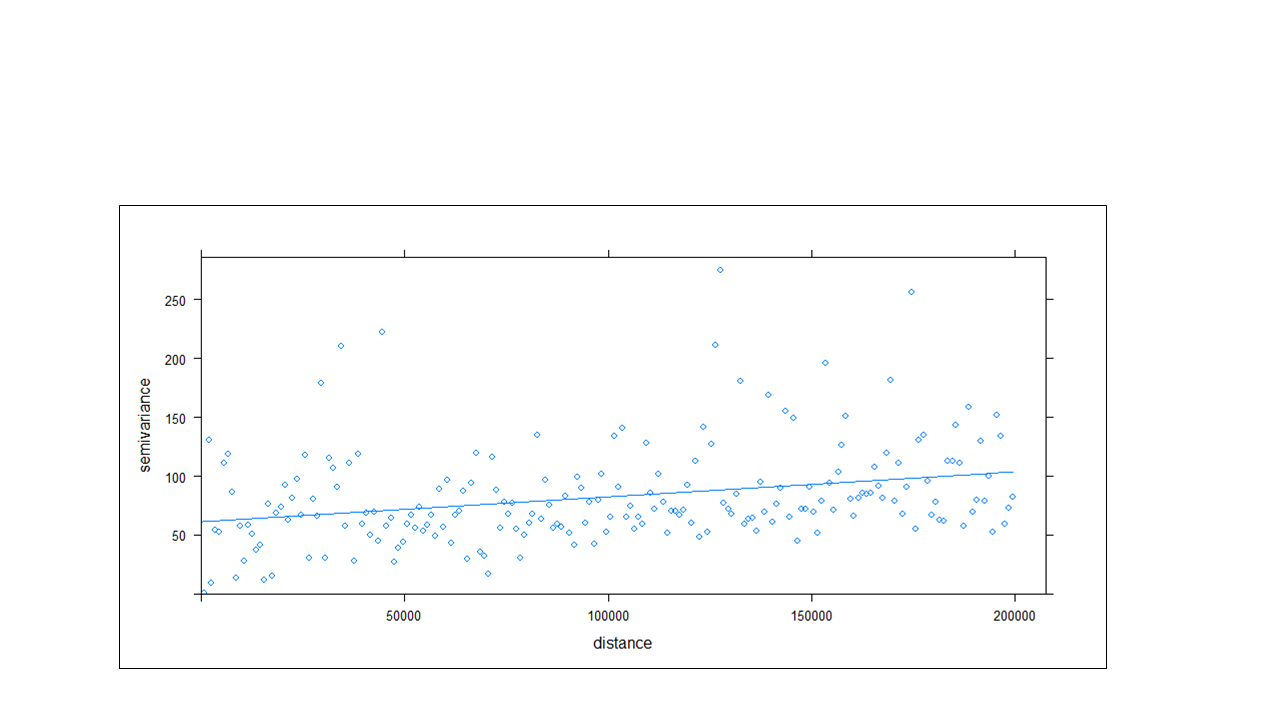

Supplement: S9 Fig — Spherical model with a partial sill of 75; a range of 50,000m; and a nugget of 10. (TIF) [file pntd.0011896.s012.tif]

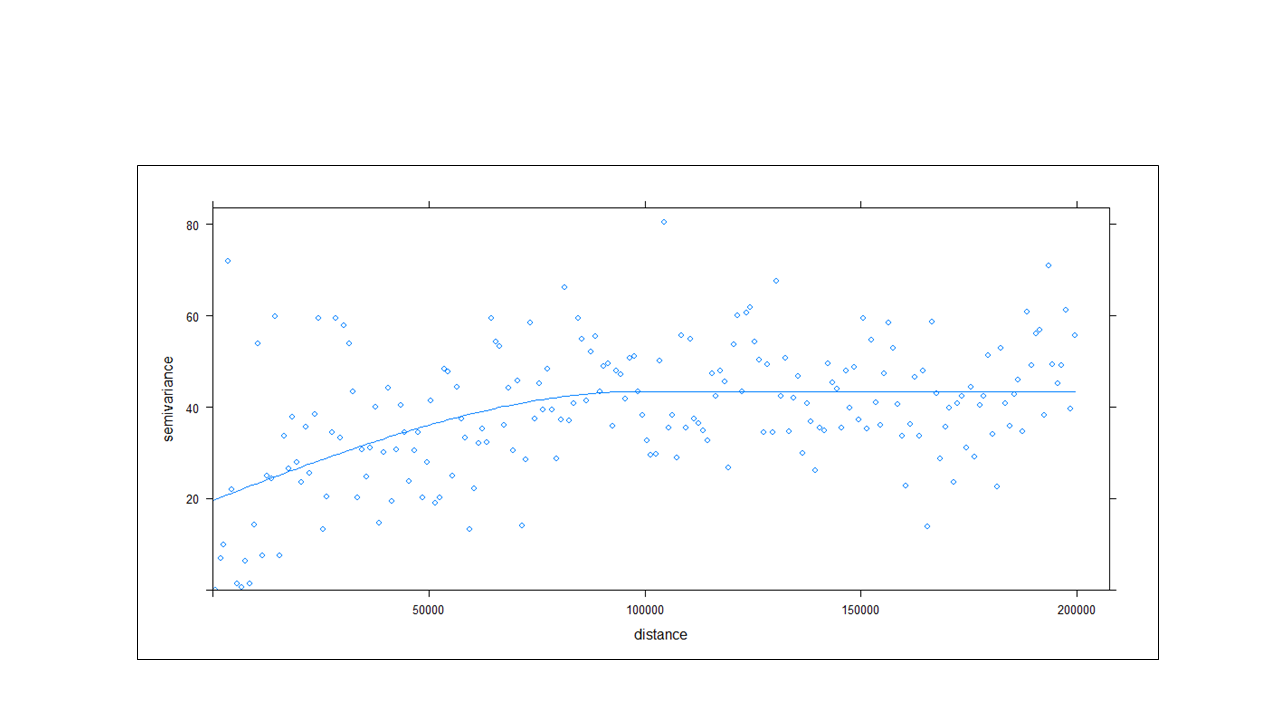

Supplement: S10 Fig — Spherical model with a partial sill of 40; a range of 90,000m; and a nugget of 1. (TIF) [file pntd.0011896.s013.tif]
