# Supplementary material for: Enabling targeted mass drug administration for schistosomiasis in north-western Tanzania: Exploring the use of geostatistical modeling to inform planning at sub-district level
Source: PLoS Negl Trop Dis. 2024 Jan 16;18(1):e0011896. doi: 10.1371/journal.pntd.0011896 (PMC10817176; doi:10.1371/journal.pntd.0011896)
Supplement: Attachment — Submitted filename: Review of PNTD D-23-00690.pdf [file pntd.0011896.s014.pdf]

Review of PNTD-D-23-00690

Schistosomiasis transmission and infection exhibits focal distribution patterns, and high variability on local (subdistrict/ward) levels. This raises many challenges for MDA control programs that rely on district level assessment, whereby many communities could be under- or over-treated. One way to address this problem is precision mapping on sub-district scale, a subject of active current research.

The authors propose another approach for accurate sub-district prediction based on geostatistical modeling. Specifically, they use zero-inflated Poisson distribution of infection burden on local ward level, and regression kriging method for geospatial prevalence assessment in unobserved wards. They identify the essential demographic, geo-spatial, environmental covariates for the model, and apply it to the dataset collected in Lake Zone endemic areas of Tanzania.

The key feature, which motivated model development, is its potential to accurately predict granular burden distribution from sparse (sub-district) sampling. Resource limitations for screening and treatment pose major challenge for many control programs, and the model suggests a novel way to address this issue. Indeed, their analysis shows a marked reduction of over and under-treatment in the study area through a more targeted approach to MDA, based on model predictions.

Overall, the study makes a valuable contribution to the ongoing effort on precision mapping within scarce resources and limited surveillance capacity.
Couple **questions** came to mind while reading it

1. One of limitations mentioned by the authors is the community status, which in the current version represents a precontrol (baseline) state of the study area, and a need to extend it to other intermediate MDA-control stages, as well as other geographic areas and settings. I wonder how the authors envision such extensions. What kind of data and modeling tools would be required?
2. Another question concerns mixed Sh-Sm infections. The study area has shown two species mostly segregated with some overlap. I wonder how one should deal with cases (communities) with strong overlap and a sizable fraction of mixed infections. What would ZIP represent in such cases, a combined burden distribution by two species? It seems the paper analysis separated Sh and Sm (KK and urine filtration). How two diagnostics could be combined? It needs some clarification.

Minor typos:

Lines: 306, 334
