# Supplementary material for: Enabling targeted mass drug administration for schistosomiasis in north-western Tanzania: Exploring the use of geostatistical modeling to inform planning at sub-district level
Source: PLoS Negl Trop Dis. 2024 Jan 16;18(1):e0011896. doi: 10.1371/journal.pntd.0011896 (PMC10817176; doi:10.1371/journal.pntd.0011896)
Supplement: Attachment — Submitted filename: PNTD-D-23-00690 comments LC.pdf [file pntd.0011896.s015.pdf]

General comments

This paper by Mathewson *et al.* aims to address the relevant and important question of how precision-mapping, aided by geospatial analysis, can contribute to more cost-efficient use of limited resources in SCH deworming programs. I advise major revision as I have a number of concerns about the used methodology and inconsistencies in the results (see specifics below).

Specific comments

Please clarify how the Kato-Katz was conducted (single/duplicate slide? was the stool homogenised before slide preparation?).

Please clarify how the two-sample t-tests were applied to check for representativeness of sampled areas in terms of ecological conditions. Was this a comparison of values at pixel level or at the level of wards (e.g., mean of pixel-level values in a ward)? Also, please clarify how one dealt with pixel-level values being correlated and the varying number of pixels per ward.

Please justify the use of a Poisson likelihood (zero-inflated or not) to fit to data on the number of positive cases in samples of 60 children. Why not a binomial model (zero-inflated or not)?

Please justify why the spatial correlation in the data was captured in a second separate model, conditional on the ZIP model estimates. These two models should not be hard to integrate and estimate in one go. Also, the variable selection procedure for the ZIP model currently does not account for spatial correlation, meaning that the standard errors for the model coefficients are off (i.e., probably underestimated). Why not avoid this and fit a single model with zero-inflation and spatial correlation? This also avoids having to explain exactly how one adds the kriging regression results to the ZIP model predictions (which is not explained, and which I find hard to envisage as the ZIP model is a mixture model). Granted, Stata may not be up to the task, but R surely is. Also, please clarify how the spatial data were constructed / analysed (exact geolocation of each school, kid, sample?). Fig 4 presents model estimates at the centroid of each ward, which to some might suggest that kriging was applied to the ward-level estimate, located at the centroid.

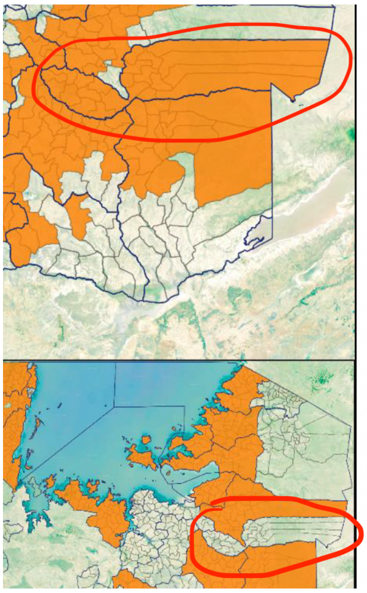

Two variables were excluded because of high collinearity. Please clarify whether the two variables were collinear with one another or with other variables. Please also justify then why these two variables were excluded and not the other variables that they were collinear with. Also, please clarify what the abbreviation "VIF" means.

← Fig 6 (left): I don't follow why a district-level aggregation of model predictions can result in a district not being eligible for treatment (prevalence <10%) while according to the ward-level results, all wards in that district are eligible for treatment (prevalence > 10%). Also, this pattern is inconsistent with Fig 7 (below), where there apparently is an "over-treated"

ward, which according to the cluster-level results in Fig 6 deserves treatment!

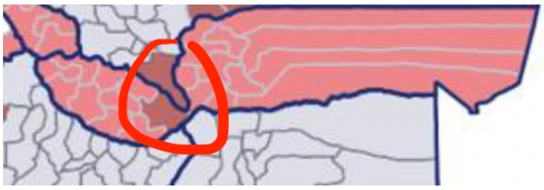

← Fig 7

Is this perhaps an artifact as a result of an error in data processing, or perhaps due to not considering uncertainty in the classification of prevalences being above or under 10%?

Minor points

- Line 221: *"The density of the snail intermediate hosts was found"* suggests that this was found in the study, but the authors probably mean to say that this *"has been found"* previously. Same sentence, but later, *"have been shown"* is used (plural), but this doesn't fit the subject of the sentence *"the density of ..."* (singular).
- Lines 306 and 334: corrupted cross-references (*"Error! Reference source not found"*).
- Table 2: the line numbers appear within the table. Also, it took me a bit of effort (more than usual) to understand the numbers in the column "Est.". Some are prevalences in the total sample while others are prevalences/proportions among positives.
- Table 3: a mix of n-dashes and m-dashes is used in the confidence intervals. Consider using commas instead as some of the numbers are <0.
- General: the authors use the term "MDA" where they actually mean "PC" (preventive chemotherapy). MDA is a specific form of PC where the majority of the community is targeted, like in onchocerciasis and LF control programs. The correct terminology for (often school-based) SCH and STH deworming programs is "targeted PC" or "school-based PC", as per WHO custom/nomenclature.
