# Supplementary material for: Enabling targeted mass drug administration for schistosomiasis in north-western Tanzania: Exploring the use of geostatistical modeling to inform planning at sub-district level
Source: PLoS Negl Trop Dis. 2024 Jan 16;18(1):e0011896. doi: 10.1371/journal.pntd.0011896 (PMC10817176; doi:10.1371/journal.pntd.0011896)
Supplement: Attachment — Submitted filename: Response to Reviewers_mathewson.docx [file pntd.0011896.s016.docx]

Dear Reviewers,

Thank you for taking the time to review our manuscript, and for providing the very useful feedback that has allowed to evaluate different parts of our manuscript and make substantial revisions. Please find your comments addressed in the authors response column the right of your comment. In the response, we furthermore refer to changes made in the manuscript and the line number where those changes can be found. These lines specifically refer to the revised manuscript with track changes.

We hope that you will find your corrections and requests for clarity adequately addressed, and we will be happy to respond to any further comments and questions going forward.

Sincerely,

Jake Mathewson

| **Reviewer Comment** | **Author Response** |
| --- | --- |
| *From Reviewer PDF (*David Gurarie) | |
| One of limitations mentioned by the authors is the community status, which in the current version represents a pre-control (baseline) state of the study area, and a need to extend it to other intermediate MDA-control stages, as well as other geographic areas and settings. I wonder how the authors envision such extensions. What kind of data and modeling tools would be required? | This is an integral point, thank you for bringing this up. The following was added to the discussion regarding the selection of covariates. (Starting on line 557)  “Reported drug coverage data from MDA campaigns have been shown to be highly variable in accuracy, often incorrectly describing the proportion of the target population that received the drug(45–48). Furthermore, this Coverage data is reported at the district level, which even when accurate, may not homogenously describe the wards within the districts. As the purpose of the model was to be able to enable targeted coverage based upon ecological and socio-demographic risk for schistosomiasis infection, the researchers strived to use covariates with data sources available at or below the ward level. Finally, none of the districts within the Lake Zone regions in the study area were found to have completed the 5 rounds of effective MDA that would provide an indication for not recommending further MDA campaigns per WHO guidelines.” |
| Another question concerns mixed Sh-Sm infections. The study area has shown two species mostly segregated with some overlap. I wonder how one should deal with cases (communities) with strong overlap and a sizable fraction of mixed infections. What would ZIP represent in such cases, a combined burden distribution by two species? It seems the paper analysis separated Sh and Sm (KK and urine filtration). How two diagnostics could be combined? It needs some clarification. | Thank you for bringing up this important point. The separate and anonymized data sets for Sh and Sm infections did not allow the analysis team to identify whether infected individuals tested positive for both types of SCH infection, or whether all Sh and Sm infections occurred in separate children. We do know, that from proportions of each strain were present in some areas, requiring treatment for both Sh and Sm (Kagera region). Fortunately, per WHO guidelines, such areas would incur the same intervention as areas with infection of a single strain exceeding the threshold for treatment, provided that the infection did not exceed 50% of tested children. The two ZIP and regression kriging (RK) models examined each strain and their relationships with ecological covariates independently from the other strain, where Sh model do not account for Sm infections and vice versa. Therefor each ZIP and RK model represent either Sh burden or Sm burden, not combined. We hope that this is adequately explained in line 286 of the methods section.  We agree that in future studies it could be very interesting to examine case based data to take a closer look at infection rates with both strains, as well as in studies with explanatory models to look at relationship between areas with the two infections. |
| *From Reviewer PDF (*Luc Coffeng) | |
| Please clarify how the Kato-Katz was conducted (single/duplicate slide? was the stool  homogenised before slide preparation?). | Thanks for bringing this up, this is an important point for assessing the quality of the egg counting process that was not previously clear in the methods.  During KK, stool did not undergo homogenization before being added to the slides. Instead, samples of the stool were taken from 4 different areas of the stool to account for different egg concentrations and then added to duplicate slides using thick smear. A third evaluator was used to make a blinded assessment of 20% of all positive and negative results to provide QA to both urine and stool samples.  The methods section has been modified to show more detail of this process (starting on line 221), and the references to the published precision mapping study remain in place should the reader want more info about methods of the school sampling. |
| Please clarify how the two-sample t-tests were applied to check for representativeness  of sampled areas in terms of ecological conditions. Was this a comparison of values at  pixel level or at the level of wards (e.g., mean of pixel-level values in a ward)?  Also,  please clarify how one dealt with pixel-level values being correlated and the varying  number of pixels per ward. | The T-test were done comparing the observed ecological conditions in wards which did not have schools that were sampled, with those which do have schools. Each of the covariates used in the model were aggregated to the ward level by taking the mean of all observations within a single ward. As the different covariates were derived from different sources with different spatial support (spatial points, grid, adm3 sampling units) they were all aggregated to the largest common denominator (wards). We agreed that this could have been made clearer so we added a line in the methods section (Line 275).  The aggregation of covariates derived from remotely sensed (gridded) raster data to a ward implicitly addresses the risk of spatial autocorrelation and hence a lack of independence in the observed values at high spatial resolution. While aggregation is likely to reduce the risk of lack of independence, the same issue could arise when ward level observations are used, with an increased risk of a type I errors. As the t-tests showed no significant differences, leading to the conclusions that sampled ward and non-sampled wards show variation in ecological conditions, type I errors did not occur. |
| Please justify the use of a Poisson likelihood (zero-inflated or not) to fit to data on the  number of positive cases in samples of 60 children. Why not a binomial model (zeroinflated  or not)? | Thanks for raising this point. The short answer is, that while we acknowledge that use of poisson likelihood may not be perfect from a theoretical perspective, the proportion of positive cases was relatively low and the mixture model (zero inflated poisson model) showed the best fit to the observed data (over then binomial and other models that were also fitted). |
| Please justify why the spatial correlation in the data was captured in a second separate  model, conditional on the ZIP model estimates. These two models should not be hard  to integrate and estimate in one go. Also, the variable selection procedure for the ZIP  model currently does not account for spatial correlation, meaning that the standard  errors for the model coefficients are off (i.e., probably underestimated). Why not  avoid this and fit a single model with zero-inflation and spatial correlation? This also  avoids having to explain exactly how one adds the kriging regression results to the  ZIP model predictions (which is not explained, and which I find hard to envisage as  the ZIP model is a mixture model). Granted, Stata may not be up to the task, but R  surely is. | We used regression kriging as opposed to econometric models (e.g. BYM) to emulate a methodology which has been well established through a series of other studies (DOI’s below). These studies show how geostatistical models (regression kriging), are used to predict a variable of interest in areas where no observations were made.  As the objective of our study is to create a predictive model (as opposed to explanatory) to produce ward level estimates of schistosome prevalence and interpolating to areas where the outcome of interest was not observed, geostatistical methods are a widely accepted method (see application examples below).  Theory  https://www.sciencedirect.com/science/article/abs/pii/S0016706103002787  <https://www.sciencedirect.com/science/article/abs/pii/S0098300407001008>  <https://www.taylorfrancis.com/books/mono/10.1201/9781315188492/model-based-geostatistics-global-public-health-peter-diggle-emanuele-giorgi>  Application  https://www.thelancet.com/journals/laninf/article/PIIS1473-3099(15)00067-5/fulltext  <https://pubmed.ncbi.nlm.nih.gov/19490724/>  https://parasitesandvectors.biomedcentral.com/articles/10.1186/s13071-020-04413-7 |
| Also, please clarify how the spatial data were constructed / analysed (exact  geolocation of each school, kid, sample?). Fig 4 presents model estimates at the  centroid of each ward, which to some might suggest that kriging was applied to the  ward-level estimate, located at the centroid. | All analysis, including model building, were conducted using data aggregated to the ward level. Semi variograms were indeed calculated based upon ward centroid locations. Thanks for bringing up this point, we have added it to the methods of the manuscript for clarity (line 302). |
| Two variables were excluded because of high  collinearity. Please clarify whether the two variables  were collinear with one another or with other  variables. Please also justify then why these two  variables were excluded and not the other variables  that they were collinear with. | Multi-collinearity among predictor variables was examined, generally indicated by a Variable Inflation Factor (VIF) above 10. The VIF indicates the overall severity of collinearity for each predictor variable in the context of all the other predictor variables in the model, and does not provide information about the specific pairs of variables causing the collinearity. We chose to remove the variables with a VIF above 10 to be sure that multi-collinearity in the remaining set of covariates was acceptable. A mean VIF of 2.13 (range: 1.44-3.53) was observed among the remaining set of 10 covariates in the full model. |
| Also, please clarify what  the abbreviation “VIF” means. | Thanks for flagging this, I have added this into the paper. |
| Fig 6 (left): I don’t follow why a district-level  aggregation of model predictions can result in a district  not being eligible for treatment (prevalence <10%)  while according to the ward-level results, all wards in  that district are eligible for treatment (prevalence >  10%). | Thank you for bringing up this important point. Given the limited capacity of programs to comprehensively survey these areas, no schools were sampled in this district during this study.  With no schools sampled within this district, three would be no indication for the requirement of PC treatment here in a conventional district level approach. The model however, has estimated that each individual ward within the district would have a prevalence above 10% warranting treatment.  I have attempted to make this clearer in the Figure. 6 description. We have also added a few lines (line 495) in the discussion about this to highlight this as a potential advantage of using geostatistical models, being able to predict in areas where no mapping was conducted. |
| Also, this pattern is inconsistent with Fig 7  (below), where there apparently is an “over-treated” ward, which according to the cluster-level results in Fig 6 deserves treatment! | See below: |
| Fig 7  Is this perhaps an artifact as a result of an error in data processing, or perhaps due to  not considering uncertainty in the classification of prevalences being above or under  10%? | Thank you for catching this detail! This was indeed the result of a data processing issue caused by matching ward names (of which there are several duplicates) instead of figure IDs. When these were instead joined using figure IDs it corrected several incorrect shadings of wards. This is reflected now in the updated Figure 7. |
| Line 221: *“The density of the snail intermediate hosts was found”* suggests  that this was found in the study, but the authors probably mean to say that this  *“has been found”* previously. Same sentence, but later, *“have been shown”* is  used (plural), but this doesn’t fit the subject of the sentence *“the density of …”*  (singular). | Thanks for flagging this, it is an important point. These were adjusted to better reflect that they were findings in literature and not part of our study. |
| Lines 306 and 334: corrupted cross-references (*“Error! Reference source not*  *found”*). | Thank you for flagging these, they have been adjusted to reflect the correct tables and figures. |
| Table 2: the line numbers appear within the table. Also, it took me a bit of  effort (more than usual) to understand the numbers in the column “Est.”. Some  are prevalences in the total sample while others are prevalences/proportions  among positives. | Thanks for pointing this out, this table did indeed have elements that needed to be clarified. Please see the new version in the text which has been made more intuitive. |
| Table 3: a mix of n-dashes and m-dashes is used in the confidence intervals.  Consider using commas instead as some of the numbers are <0. | Thank you for this suggestion, this change was indeed implemented for clarity in Table 3. |
| General: the authors use the term “MDA” where they actually mean “PC”  (preventive chemotherapy). MDA is a specific form of PC where the majority  of the community is targeted, like in onchocerciasis and LF control programs.  The correct terminology for (often school-based) SCH and STH deworming  programs is “targeted PC” or “school-based PC”, as per WHO  custom/nomenclature. | Thanks for flagging this, there were indeed some areas of the publication that were made clearer by changing MDA to PC. It was convenient to mainly refer to MDA in the discussion about |

--------------------------------------------Reviewers response to questions in the Email------------------------------

| From Reviewer One | |
| --- | --- |
| (Methods) The authors state in the Discussion that not including past MDA coverage in the model is a limitation. However, they don't explain why this couldn't have been included, since surely this data would have been available from the schools that were surveyed (e.g., length of time since last MDA as a variable)? - I am not a statistician, so cannot comment critically on the methods that were used. | This is an integral point, thank you for bringing this up. The following was added to the discussion regarding the selection of covariates. (Starting on line 557)  “Reported drug coverage data from MDA campaigns have been shown to be highly variable in accuracy, often incorrectly describing the proportion of the target population that received the drug(45–48). Furthermore, this Coverage data is reported at the district level, which even when accurate, may not homogenously describe the wards within the districts. As the purpose of the model was to be able to enable targeted coverage based upon ecological and socio-demographic risk for schistosomiasis infection, the researchers strived to use covariates with data sources available at or below the ward level. Finally, none of the districts within the Lake Zone regions in the study area were found to have completed the 5 rounds of effective MDA that would provide an indication for not recommending further MDA campaigns per WHO guidelines.” |
| (Results) It is surprising and counterintuitive that the model predicts that water source improvement and reduced open defecation were associated with prevalence of urinary schistosomiasis. The authors should comment on this. | Thanks for bringing up this point, indeed it seems counterintuitive that the logit model shows an association between open defecation and urogenital schistosomiasis.  As this is not an explanatory model, the purpose was not to interpret any of the individual covariates and their univariate correlations to the number of cases. These should be interpreted within the light of the full model, including the logit and the count model, and assessed on its predictive accuracy.  This is why we try to avoid commenting in the publication further on these individual associations, to avoid the false interpretation of the covariates, as one would do if we had presented an explanatory model.  While it is not our objective to quantify risk relations *per se*, it is important that the model presented makes sense from an epidemiological point of view and is compared to existing evidence regarding ecological risks of schistosomiasis. We therefore present the ZIP model results, before presenting the complete geostatistical model which furthermore includes kriging, for readers to understand how the model was built. |
| (Conclusions) The authors state that their results effectively predict ward-level parasite prevalence, yet no validation of their model was performed. That is, the predicted prevalence in non-surveyed wards wasn't then tested by conducting actual prevalence testing in those wards to confirm if the model's prediction was accurate. Therefore, the statement that the model "effectively predicted" prevalence is an over-ambitious one and does not reflect the actual data (which are speculative). | Thanks for pointing this out, this indeed gave the impression that these results had been validated which have not yet had the opportunity to do. We have no intention to demonstrate that this has yet been validated, and have removed ‘effectively’ from the text. |
| - It would have been interesting for the authors to have included even a rough estimate of the cost savings that would have been realized had MDA been restricted to the model-predicted higher prevalence wards. | Indeed this would have been very interesting to have discussed and would have added an additional dimension to the study. A cost effectiveness analysis is a very good idea, but beyond the scope of this study.  We did not feel suited to make estimation about costs of switching to ward level treatment given all that we still do not know about how targeted schistosomiasis MDA would be delivered, and also what this would mean for uncoupling the delivery of different disease MDA campaigns which has been shown to reduce costs (ex. Soil transmitted helminths with schistosomiasis). We think this will be an extremely important next step and have added a few lines in the recommendation. Thanks for bringing it up. |
| Abbreviations should be defined in the text, not in a separate table. Also, some abbreviations in the text aren't defined anywhere in the manuscript (e.g., "IU"). | This was adjusted anywhere we can find the abbreviations in text (IU), thanks for this reminder. |
| Introduction, lines 111-113: a statement is made that prevalence is increasing, but absolute numbers of cases are given as supporting evidence instead of percentages, which would be more appropriate. | Thanks for highlighting this important point, given the rapid population growth in Tanzania in the past decade, whole numbers do not offer easy to interpret picture of schistosomiasis burden. We have adjusted the text and references accordingly. |
| Introduction, line 131: delete statement about Tanzania having the 2nd highest burden of schistosomiasis as this is repeated from above. | Thank you for catching this repeat, it has indeed been removed from the paper. |
| Methods, line 246: the word "ZIP" appears here inappropriately. | Thank you for catching this, it has been removed from the paper. |
| Results, line 301: should be "intestinal schistosomiasis" instead of just "intestinal". | Thank you for that comment, I agree that it makes it more clear, this has since been added. |
| Results, line 302: should be "further inland" not "further inlands" | Thanks for catching this grammatical error, it has been changed. |
| Results: several of the Table and Figure references appear as "Error! Reference source not found". Recommend removing the hyperlinks | Thank you for flagging this, these hyperlinks have been removed in place of the table and figure names. |
| Results, lin 336: the second instance of "prevalence" in this sentence should be deleted | Thank you for catching this, it was deleted accordingly. |
| Figure 5 appears twice in the document | Indeed it does! Thanks for catching this, the extra figure was removed. |
