# Supplementary material for: Enabling targeted mass drug administration for schistosomiasis in north-western Tanzania: Exploring the use of geostatistical modeling to inform planning at sub-district level
Source: PLoS Negl Trop Dis. 2024 Jan 16;18(1):e0011896. doi: 10.1371/journal.pntd.0011896 (PMC10817176; doi:10.1371/journal.pntd.0011896)
Supplement: Attachment — Submitted filename: Response to reviewers_round2.docx [file pntd.0011896.s017.docx]

Dear Reviewers,

Thank you for taking the time to make this second revision of our manuscript, and for providing further feedback to our responses and changes. As in the first round, these suggestions have helped us to improve our manuscript.

Please find your comments addressed in the authors response column the right of your comment. In the response, we furthermore refer to changes made in the manuscript and the line number where those changes can be found. These lines specifically refer to the revised manuscript with track changes.

We hope that you will find your corrections and requests for clarity adequately addressed, and we will be happy to respond to any further comments and questions going forward.

Sincerely,

Jake Mathewson

| **Reviewer 1** | |
| --- | --- |
|  | |
| The authors removed the word "effectively" from the statement that their model can be used to predict the prevalence of schistosomiasis, but the point of the prior comment was that there has been no validation of the model's prediction (i.e., comparing predicted prevalence against real data). It was the entire statement that is problematic, not just the word "effectively". Please comment. | Thank you for this comment. I see now more clearly what you had initially suggested. It was indeed not our intention to suggest that the model estimates had been validated (as they have not). The point that we were trying to state was that these open source covariates could be used to generate prevalence estimates at a sub-district, ward level. Agreeing that the initial text was misleading, we have made the following change in the latest version:  [Line 453]  “ The study also found that routine and publicly available data such as population density, stunting rate, distance to a lake, terrain ruggedness, vegetation, wetness, and use of improved water sources can be used to generate prevalence estimates of both types of schistosomiasis at a sub-district, ward level.” |
| There are several spelling errors in the newly added text (e.g., "Tanzania" and "because") are misspelled. | Thank you for catching these errors within the tracked changes. We have done a more careful and comprehensive revision of the manuscript and made corrections where necessary, including these examples. |
|  |  |
| **Reviewer 3** | |
| The authors now better explain how the Kato-Katz (KK) was performed. However, from their reply to the comments, it seems they prepared quadruple slides based on a single faecal sample per person, whereas the text in the manuscript suggests that two faecal samples were collected per person and that two slides were prepared per faecal samples ("two sets of duplicate slides"). Please clarify. Also, in their reply to the reviewer comments, the authors clarify that stools were not homogenised (which is important, as they acknowledge) but this is not mentioned in the revised manuscript (with tracked changes). | Thank you for this point, I see how this could still be interpreted ambiguously in the way that it is written. By two sets of duplicate slides I did indeed intend to imply that it was done on 4 slides.  I have made further adjustments in the tracked changes version to try to make this clearer:  [line 213]  “The Kato Katz technique was used for intestinal schistosomiasis, with processed samples prepared on four slides (two sets of duplicate slides) using thick smear for two technicians to evaluate. Thick smears for each of the four slides were prepared using distinct areas of the stool sample to account for a potentially homogeneous distribution of eggs within fecal samples.” |
| I repeat my earlier comment about the use of a separate ZIP model and posthoc kriging, and the problem this approach poses for variable selection. In their reply, the authors explain why they use a geospatial approach (which I agree is appropriate but is not the core of my question) and that they intended to "emulate" the established geospatial approach, such as described by Giorgi, Diggle, etc (which I'm familiar with). The problem is that this emulation approach leads to underestimation of standard errors on coefficients in the first step (the ZIP model), which is also used for variable selection. If the authors would instead use the established geospatial approach itself (rather than emulate it in two steps) and base their variable selection on that, they may end up with fewer predictors in the model (because of more conservative and appropriate estimation of standard errors of coefficients). | Thanks for reiterating this comment, it is an important point. We have taken the approach as set out by Hengl et al (2007) Cressie (1993) to generate unbiased predictions. For the trend model (e.g. ZIP), initial variable selection was done based on existing knowledge of factors associated with schistosomiasis prevalence from published literature. To prevent multicollinearity while fitting models, single covariates were excluded before based high pairwise cross-correlations. The final set of covariates was based on the overall predictive accuracy of a model rather than the partial fit. Covariates that were included therefore maximized predictive accuracy.  We have added the following to the Methods section: Line[231]  “Covariates were only excluded if there were high degrees of collinearity or if they reduced the goodness of fit based on the AIC, not according to levels of significance in univariate analysis.”    We have furthermore acknowledge that the modeling approach may have led to an underestimation of SE. We have noted this in the limitations.  Line[537]  “Additionally, the modeling approach used may have led to an underestimation of standard errors in the ZIP model, however, since the model did not exclude covariates based on their univariate associations, but rather the overall predictive accuracy of the model, it is unlikely this would have altered the variable composition of the geostatistical models. Because of this, care should be taken in interpretation of associations of individual covariates with schistosomiasis prevalence.” |
| Figure 6: it is now clear that the left panel is based on a crude district-level aggregation of survey results, while the right panel is based on ward-level model predictions. As one of the main focus points of the paper is ward-level vs. district-level policy, a logical intermediate step in Figure 6 would be to present a middle panel with district-level results based on aggregated model-predictions (instead of the raw data). The authors could even consider adding a fourth panel indicating ward-level results based on raw data, indicating for which wards no inference would be possible as there was not data. This would be a useful illustration for the reader to appreciate which benefits originate from which part of the approach: the use of a geospatial model and the consideration of ward vs. districts as unit of aggregation. | Thank you for critically thinking along with the best way to convey this figure.  For the first suggestion: I am apprehensive to include a map with district level treatment based on the aggregated ward level estimates, mostly because I think that it may confuse the reader about our primary intention, which is to make ward level estimates and to allow for targeted ward level treatment in place of district level treatment.  For us, the key benefit of providing granular model estimates is to enable targeted (ward) level treatment, and not to use the model estimates as a separate method of continuing to target whole districts. I fear that a district level representation of the model results may cloud that message.  We did, however, think your second suggestion to include ward based planning based solely on observed data is interesting and highlights another key point of using a model: that without using some kind of modeling you have very sparse data with which to make estimates. For formatting purposes, we tried to create a 3^rd^ panel with the ward level, seen below:    However again, we felt like it distracted from the main benefit of this figure, a side by side comparison of the distinct geographical areas treated with the two different methodologies. Although a good suggestion, we ultimately decided to leave the figures the same for the most cohesive narrative (Link of Fig 6 to Table 4). |
